# Predictive value of composite nutritional indicators geriatric nutritional risk index and controlling nutritional status for mortality risk in early-onset cancer survivors

**DOI:** 10.3389/fnut.2025.1598043

**Published:** 2025-08-07

**Authors:** Wenyang Li, Zhixuan Mou, Songtao Yu, Chenkai Zhang, Haonan Qi, Guiyu Wang

**Affiliations:** ^1^Department of Colorectal Surgery, The Second Affiliated Hospital of Harbin Medical University, Harbin, China; ^2^Department of Pediatric Surgery, The Sixth Affiliated Hospital of Harbin Medical University, Harbin, China; ^3^Department of Colorectal Surgery, Zhejiang Cancer Hospital, Hangzhou Institute of Medicine, Chinese Academy of Sciences, Hangzhou, Zhejiang, China

**Keywords:** GNRI, CONUT, nutrition, early-onset cancer, inflammation

## Abstract

**Background:**

Malnutrition represents a critical determinant of adverse clinical outcomes and substantial disease burden in cancer patients. Despite the established prognostic value of Geriatric Nutritional Risk Index (GNRI) and Controlling Nutritional Status (CONUT) as composite inflammation-immune-nutrition indices in elderly cancer patients, their utility in early-onset (EO) cancer survivors remains unclear.

**Methods:**

This retrospective study evaluated GNRI and CONUT for predicting mortality in 3,273 early-onset (EO) cancer survivors, with a development cohort (*n* = 2,814) from NHANES (1999–2018) and a validation cohort (*n* = 459) from the Second Affiliated Hospital of Harbin Medical University (2011–2020). Patients were stratified by GNRI (< 98 vs. ≥ 98) and CONUT (≥ 2 vs. ≤ 1) and grouped into composite risk categories: High-risk (GNRI < 98 + CONUT ≥ 2), Moderate-risk (GNRI < 98 + CONUT ≤ 1 or GNRI ≥ 98 + CONUT ≥ 2), and Low-risk (GNRI ≥ 98 + CONUT ≤ 1).

**Results:**

In the development cohort, GNRI < 98 and CONUT ≥ 2 independently predicted elevated risks of all-cause mortality (HR = 3.36, 95%CI = 2.69–4.19, *P* < 0.001), cancer-specific mortality, and non-cancer mortality. High-risk patients exhibited the poorest survival outcomes compared to Low-risk (all-cause mortality HR = 3.36, *P* < 0.001). Kaplan-Meier analysis confirmed worse prognosis in GNRI < 98, CONUT ≥ 2, and High-risk groups across all mortality endpoints. Validation cohort results aligned with these findings, reinforcing the prognostic significance of composite nutritional risk stratification.

**Conclusion:**

This study is the first to validate GNRI and CONUT as effective composite inflammation-immune-nutrition indices for identifying high-risk EO cancer survivors. Composite stratification combining both indices enhances multidimensional inflammation-immune-nutrition risk assessment, offering a practical framework for prognostication and personalized care in this population.

## Introduction

In recent years, although the incidence and mortality rates of some cancers have declined significantly with economic and medical advancements, cancer remains a major global health concern (1–5). And the incidence of early-onset (EO) cancer, defined as cancer diagnosed in individuals under 50 years of age, has shown a concerning upward trend globally (6–8). EO is associated with aggressive tumor biology and poorer clinical outcomes compared to elderly cancer patients (9). Among the multifactorial contributors to its prognosis, malnutrition stands out as a critical and modifiable risk factor. Previous studies have demonstrated that disease-related malnutrition affects 40–80% of cancer patients and is strongly linked to adverse clinical consequences, including higher complication rates, diminished treatment response, impaired quality of life, and increased healthcare expenditures (10). Therefore, scientific evaluation of cancer patients’ malnutrition risk and inflammation-immune-nutrition status is crucial for optimizing clinical management and improving prognosis.

The primary objective of malnutrition screening in cancer patients is to identify individuals at nutritional risk as the critical first step in the nutritional care process. While malnutrition screening tools like the Geriatric Nutritional Risk Index (GNRI) and Controlling Nutritional Status (CONUT) score are widely used in oncology, their application in EO cancer survivors remains unexplored despite established utility in elderly populations. Importantly, these tools should be conceptualized as integrated indexes reflecting inflammation, immune response, and nutritional derangements rather than isolated nutritional assessments. The GNRI, derived from serum albumin and body weight, and CONUT, incorporating albumin, lymphocytes, and cholesterol, collectively capture multidimensional risk pathways (11, 12). Although surveys confirm the prognostic value of nutritional risk screening in general cancer populations, EO-specific evidence is notably lacking. Furthermore, no studies have examined whether combining GNRI and CONUT enhances risk stratification in this young cohort.

This study aims to validate GNRI and CONUT as inflammation-immune-nutrition indices and malnutrition risk screening tools in EO cancer survivors using the National Health and Nutrition Examination Survey (NHANES) and hospital cohorts and provide effective screening tools for clinical practice, identify high-risk populations to initiate nutritional interventions as early as possible, and achieve favorable clinical outcomes.

## Materials and methods

### Study population

The development cohort was constructed using data from the National Health and Nutrition Examination Survey (NHANES 1999–2018), a nationally representative cross-sectional program designed to systematically evaluate the health and nutritional status of American adults and children through unbiased surveys and sampling studies. The project incorporates interviews and physical examinations, encompassing demographic information, socio-economic status, dietary habits, and health-related issues. As a population-based survey, NHANES does not systematically capture detailed cancer treatment data, nor does it include specific information on cancer diagnosis. All NHANES protocols were approved by the Centers for Disease Control and Prevention National Center for Health Statistics Ethics Review Board.

**FIGURE 1:**
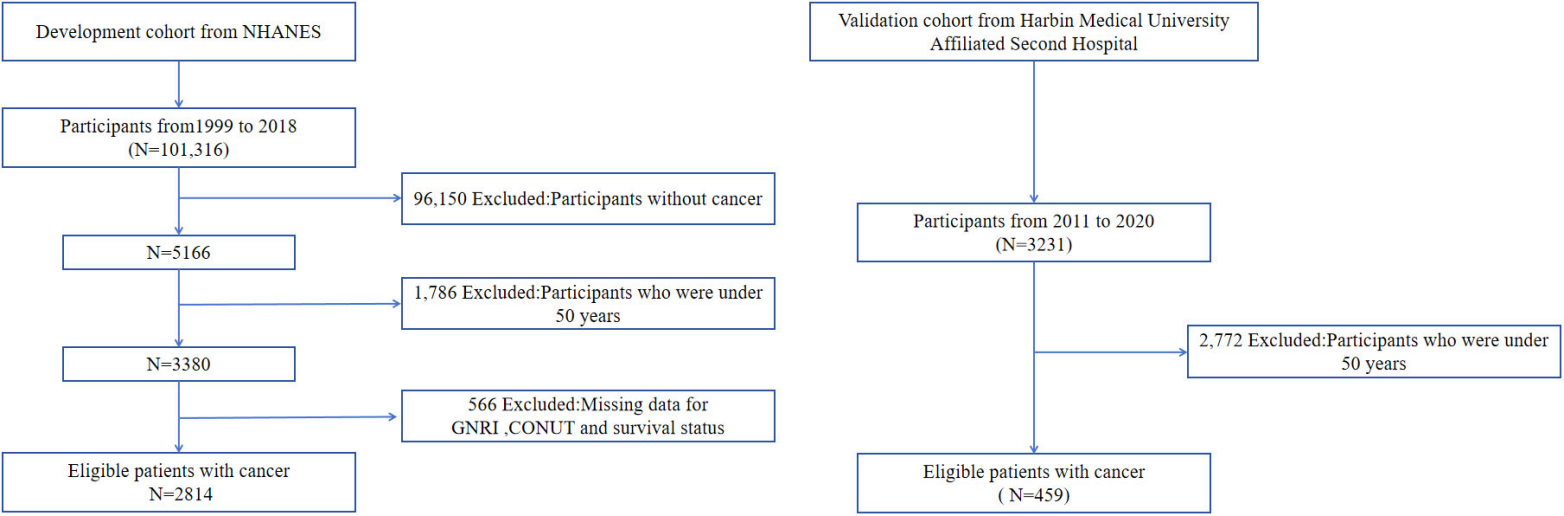
Flow chart.

We initially screened 3,380 EO cancer survivors from the NHANES database and excluded participants with unknown survival status and follow-up time (*n* = 566). Ultimately, 2,814 cancer survivors were included in this study. The validation cohort was sourced from the Second Affiliated Hospital of Harbin Medical University, and 459 patients were included in this study based on the inclusion and exclusion criteria. Notably, cause-of-death documentation in the validation cohort was incomplete, precluding robust analysis of cancer-specific mortality in this group. Consequently, validation analyses focused exclusively on all-cause mortality outcomes. All patients received guideline-directed standard therapy for their specific cancer types. The flow chart was showed in Figure 1.

### Exposure factors and outcomes

The Geriatric Nutritional Risk Index (GNRI) was first introduced by Bouillanne et al. (11), combining laboratory data and physical measurements. Its calculation formula is GNRI = 1.489 × serum albumin concentration (g/L) + 41.7 × (actual/ideal weight). The ideal weight is calculated using the Lorenz formula. Generally, patients with GNRI < 98 are considered to have high malnutrition risk, while those with GNRI ≥ 98 are deemed to have no malnutrition.

The Controlling Nutritional Status (CONUT) score is a comprehensive tool for assessing patients’ malnutrition risk and inflammation-immune-nutrition status, based on serum albumin, lymphocyte count, and total cholesterol levels. These three indicators respectively reflect the patient’s nutritional status, immune function, and metabolic status. A cumulative score of ≥2 indicates high malnutrition risk, while a score ≤1 indicates no malnutrition.

The survival status of participants in the development cohort was obtained from the National Death Index (NDI), which records participants’ survival status, follow-up time, and cause of death. The survival status in the validation cohort was collected by researchers at the Second Affiliated Hospital of Harbin Medical University, with the cause of death data being incomplete. Cancer-specific death is defined as death from malignant tumors, excluding those who did not die or died from other causes. Non-cancer-specific death is defined as death from other causes, excluding those who did not die or died from tumors. All-cause death is defined as death from any cause during the follow-up period, irrespective of the specific cause.

In the NHANES survey, each participant was queried, “Have you ever been told by a doctor that you had cancer or malignancy of any kind?” For our study, if the answer was “Yes,” they were classified as cancer survivors. In real-world settings, patients diagnosed with malignant tumors at the Second Hospital of Harbin Medical University, with complete data and accurate follow-up information, were included in our study.

### Covariates

Based on current research and consideration of covariate data, demographic data collected from the NHANES database included gender, age, and race, which were selected as confounding factors through standardized questionnaires. In terms of laboratory data, neutrophil count, white blood cell count, and platelet count obtained from standardized blood tests were selected as confounding factors. Participants reported their own ethnic background using the categories of Mexican American, other Hispanic, non-Hispanic White, non-Hispanic Black, and other race or ethnicity, with Mexican Americans grouped together and other races as one group.

Data collected from the Second Affiliated Hospital of Harbin Medical University included age, gender, T-stage, N-stage, and TNM stage. T-stage was divided into T1-2 and T3-4 groups and N-stage classification comprised N0 and N + groups. TNM staging followed the AJCC 8th edition guidelines, categorized as stage I-II and III groups. The grouping of neutrophil count, white blood cell count, and platelet count was based on the upper reference values of standardized tests, with the upper limits for neutrophil count: 6.3 × 10^9/L, white blood cell count: 9.5 × 10^9/L, and platelet count: 350 × 10^9/L.

### Data analysis

Baseline characteristics of EO cancer survivors stratified by GNRI and CONUT categories were initially characterized. Categorical variables were presented as number + percentage and compared using chi-square or Fisher’s exact test. The cox proportional hazards regression model was utilized to calculate the hazard ratio (HR) and 95%CI of GNRI or CONUT groups with all-cause, cancer-specific, and non-cancer mortality, adjusting for confounding factors such as gender, age, race, platelet count, T-stage, N-stage, and TNM stage. While data on neutrophil count and white blood cell count (as surrogate inflammatory markers) were collected from both cohorts, preliminary analysis revealed no significant association with mortality outcomes in multivariable models (*P* > 0.05). Consequently, these variables were retained only as adjustment covariates in Cox regression models rather than primary exposures. Kaplan-Meier survival curves were employed to visually depict the survival status of different groups. Forest plots were used for subgroup analysis. All statistical analyses were performed using R software (version 4.4.1) and GraphPad Prism (version 8.0.2). A *p*-value < 0.05 was considered statistically significant.

## Results

### Baseline characteristics of study participants

The development cohort had a median follow-up of 95.5 months (range: 2–249), with 1,020 all-cause deaths (including 308 cancer-related deaths). The validation cohort had a median follow-up of 59 months (range: 2–100), with 146 all-cause deaths. This study enrolled 3,273 patients, comprising 1,635 males (50%) and 1,638 females (50%), with a median age of 19 years (9–33). The validation cohort (Second Affiliated Hospital of Harbin Medical University) consisted exclusively of EO gastrointestinal cancers. When combined with the development cohort—breast cancer (14%), cervical cancer (7.5%), non-melanoma skin cancer (15.6%), unspecified skin cancer (7.8%), and prostate cancer (15%)—the study spanned diverse early-onset cancer subtypes. Patients with neutrophil count, platelet count, and white blood cell count not exceeding the upper limits had a significantly higher proportion. In the validation cohort, T3-4 stage accounted for 86.5% of cases, and lymph node involvement was observed in 62.4% of patients. Regarding TNM staging, 60.3% of patients were classified as stage I-II (Table 1).

**TABLE 1:** Baseline clinical characteristics.

Development cohort							
Characteristics	GNRI < 98	GNRI ≥ 98	CONUT ≤ 1	CONUT ≥ 2	High-risk	Moderate-risk	Low-risk
**Age (years), *n* (%)**							
<40	307 (10.9)	2147 (76.2)	1782 (63.3)	672 (23.9)	146 (5.2)	687 (24.4)	1621 (57.6)
≥40	50 (1.8)	310 (11.1)	255 (9.1)	105 (3.7)	25 (0.8)	105 (3.7)	230 (8.3)
**Sex, *n* (%)**							
Male	185 (6.6)	1166 (41.4)	964 (34.2)	387 (13.8)	87 (3.1)	398 (14.1)	866 (30.8)
Female	172 (6.1)	1291 (45.9)	1073 (38.1)	390 (13.9)	84 (3.0)	394 (14.0)	985 (35.0)
**Race, *n* (%)**							
Mexican American	96 (3.4)	600 (21.3)	484 (17.2)	212 (7.5)	43 (1.5)	222 (7.9)	431 (15.3)
Other Hispanic	26 (0.9)	182 (6.5)	143 (5.1)	65 (2.3)	13 (0.5)	65 (2.3)	130 (4.6)
Non-Hispanic White	113 (4.0)	829 (29.4)	702 (25.0)	240 (8.4)	56 (2.0)	241 (8.6)	645 (22.9)
Non-Hispanic Black	90 (3.2)	610 (21.7)	508 (18.1)	192 (6.8)	41 (1.5)	200 (7.1)	459 (16.3)
Other	32 (1.1)	236 (8.5)	200 (7.1)	68 (2.5)	18 (0.6)	64 (2.3)	186 (6.6)
**NEU, *n* (%)**							
NEU 1	299 (10.6)	2187 (77.7)	1797 (63.9)	689 (24.5)	144 (5.1)	700 (24.9)	1642 (58.4)
NEU 2	58 (2.1)	270 (9.6)	240 (8.5)	88 (3.1)	27 (0.9)	92 (3.3)	209 (7.4)
**WBC, *n* (%)**							
WBC 1	293 (10.4)	2156 (76.6)	1741 (61.9)	708 (25.1)	146 (5.2)	709 (25.2)	1594 (56.6)
WBC 2	64 (2.3)	301 (10.7)	296 (10.5)	69 (2.5)	25 (0.9)	83 (2.9)	257 (9.2)
**PLT, *n* (%)**							
PLT 1	319 (11.3)	2291 (81.4)	1871 (66.4)	739 (26.3)	149 (5.3)	760 (27.0)	1701 (60.5)
PLT 2	38 (1.4)	166 (5.9)	166 (5.9)	38 (1.4)	22 (0.8)	32 (1.1)	150 (5.3)
**Validation cohort**							
**Characteristics**	**GNRI < 98**	**GNRI ≥ 98**	**CONUT ≤ 1**	**CONUT ≥ 2**	**High-risk**	**Moderate-risk**	**Low-risk**
**Age (years), *n* (%)**							
<40	25 (5.4)	81 (17.7)	80 (17.4)	26 (5.7)	18 (3.9)	15 (3.2)	73 (16.0)
≥40	81 (17.7)	272 (59.2)	286 (62.3)	67 (14.6)	39 (8.5)	70 (15.2)	244 (53.2)
**Sex, *n* (%)**							
Male	59 (12.9)	225 (49.0)	231 (50.4)	53 (11.5)	34 (7.4)	44 (9.6)	206 (44.9)
Female	47 (10.2)	128 (27.9)	135 (29.4)	40 (8.7)	23 (5.0)	41 (8.9)	111 (24.2)
**T stage, *n* (%)**							
T1-2	10 (2.2)	52 (11.3)	55 (12.1)	7 (1.5)	5 (1.1)	7 (1.5)	50 (10.9)
T3-4	96 (20.9)	301 (65.6)	311 (67.7)	86 (18.7)	52 (11.3)	78 (17.1)	267 (58.1)
**N stage, *n* (%)**							
N0	65 (14.2)	221 (48.2)	230 (50.1)	56 (12.2)	31 (6.7)	59 (12.9)	196 (42.6)
N+	41 (8.9)	132 (28.7)	136 (29.6)	37 (8.1)	26 (5.7)	26 (5.7)	121 (26.4)
**TNM, *n* (%)**							
I-II	62 (13.5)	215 (46.8)	227 (49.4)	50 (10.9)	28 (6.1)	56 (12.2)	193 (42.0)
III	44 (9.6)	138 (30.1)	139 (30.3)	43 (9.4)	29 (6.3)	29 (6.3)	124 (27.1)
**NEU, *n* (%)**							
NEU 1	95 (20.7)	306 (66.7)	314 (68.5)	87 (18.9)	52 (11.3)	78 (17.1)	271 (59.0)
NEU 2	11 (2.4)	47 (10.2)	52 (11.3)	6 (1.3)	5 (1.1)	7 (1.5)	46 (10.0)
**WBC, *n* (%)**							
WBC 1	91 (19.8)	320 (69.7)	327 (71.3)	84 (18.3)	51 (11.1)	73 (15.9)	287 (62.6)
WBC 2	15 (3.3)	33 (7.2)	39 (8.5)	9 (1.9)	6 (1.3)	12 (2.6)	30 (6.5)
**PLT, *n* (%)**							
PLT 1	91 (19.8)	324 (70.6)	334 (72.9)	85 (18.5)	51 (11.1)	78 (17.0)	290 (63.2)
PLT2	15 (3.3)	29 (6.3)	32 (6.9)	8 (1.7)	6 (1.3)	7 (1.5)	27 (5.9)

### Association between GNRI, CONUT, malnutrition risk, and mortality outcomes

**FIGURE 2:**
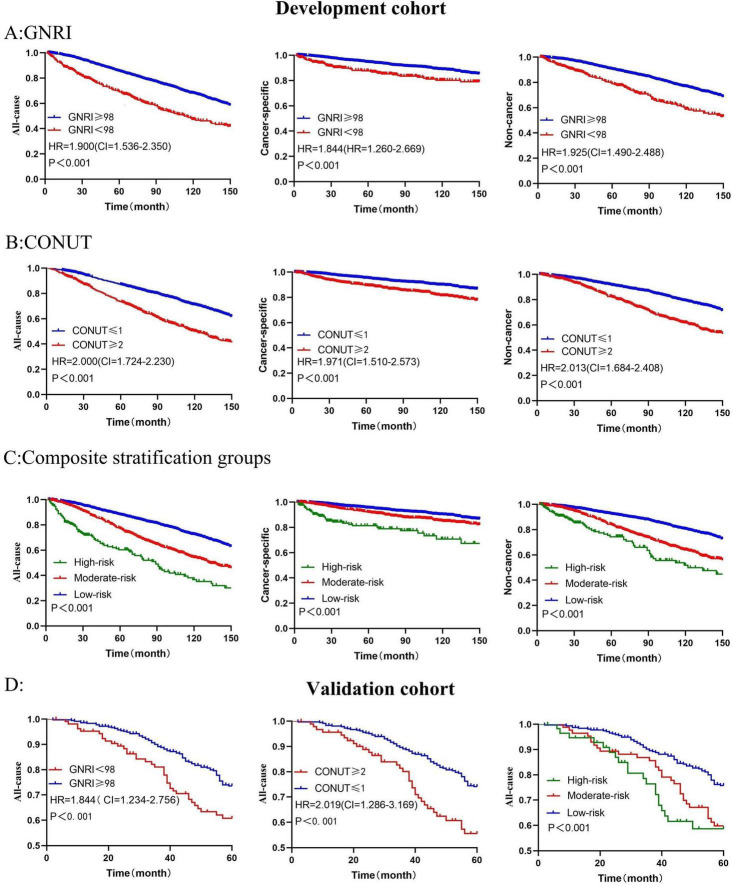
Kaplan-Meier survival curves show mortality among all groups **(A)** represents GNRI in association with all-cause, cancer-specific, and non-cancer mortality in the development cohort; **(B)** illustrates CONUT in association with all-cause, cancer-specific, and non-cancer mortality; **(C)** shows composite stratification groups in association with all-cause, cancer-specific, and non-cancer mortality. In the validation cohort, **(D)** correspond to GNRI, CONUT, and the composite stratification groups in association with all-cause mortality. *P*-values were calculated by log-rank test.

In the development cohort, univariate cox regression analysis results demonstrated that GNRI (HR = 1.940, 95%CI = 1.610–2.250, *P* < 0.001, with GNRI ≥ 98 as the reference) and CONUT (HR = 2.013, 95%CI = 1.770–2.290, *P* < 0.001, with CONUT ≤ 1 as the reference) were risk factors for the all-cause death of EO patients. These findings were also validated in the validation cohort for GNRI (HR = 1.846, 95%CI = 1.304–2.614, *P* < 0.001) and CONUT (HR = 2.032, 95%CI = 1.406–2.939, *P* < 0.001). With regard to cancer-specific death and non-cancer death, the malnutrition risks indicated by GNRI and CONUT were also risk factors for poor prognosis. Kaplan-Meier curves visually depicted the survival status of EO cancer survivors in different groups (Figure 2).

In the development cohort, GNRI and CONUT were confirmed as independent prognostic factors. After adjusting for confounding factors, compared with GNRI ≥ 98, the risk of all-cause death increased by 88% (*P* < 0.001) for cancer survivors with GNRI < 98, the risk of cancer-specific death increased by 96.3% (*P* < 0.001), and the risk of non-cancer death increased by 84.1% (*P* < 0.001). Compared with CONUT ≤ 1, the risk of all-cause death increased by 88.3% for cancer survivors with CONUT ≥ 2, the risk of cancer-specific death increased by 105.2%, and the risk of non-cancer death increased by 79.7%. In the validation cohort, compared with GNRI ≥ 98, the risk of all-cause death increased by 73.8% (*p* < 0.001) for cancer survivors with GNRI < 98, and compared with CONUT ≤ 1, the risk of all-cause death increased by 88.8% (*P* < 0.001) for cancer survivors with CONUT ≥ 2 (Table 2).

**TABLE 2:** COX proportional hazard regression analysis for GNRI and CONUT group.

Development cohort				
All-cause mortality				
Variables	Univariate analysis		Multivariate analysis	
GNRI	HR (95%CI)	*P*	HR (95%CI)	*P*
GNRI < 98	1.940 (1.610–2.250)	<0.001	1.880 (1.569–2.253)	<0.001
GNRI ≥ 98	Reference		Reference	
**CONUT**				
CONUT ≤ 1	Reference		Reference	
CONUT ≥ 2	2.013 (1.770–2.290)		1.883 (1.639–2.163)	<0.001
**Cancer-specific mortality**				
**GNRI**				
GNRI < 98	1.848 (1.364–2.503)	<0.001	1.963 (1.422–2.709)	<0.001
GNRI ≥ 98	Reference		Reference	
**CONUT**				
CONUT ≤ 1	Reference		Reference	
CONUT ≥ 2	1.938 (1.571–2.504)	<0.001	2.052 (1.604–2.625)	<0.001
**Non-cancer mortality**				
**GNRI**				
GNRI < 98	1.929 (1.578–2.357)	<0.001	1.841 (1.479–2.292)	<0.001
GNRI ≥ 98	Reference		Reference	<0.001
**CONUT**				
CONUT ≤ 1	Reference		Reference	
CONUT ≥ 2	2.027 (1.737–2.365)	<0.001	1.797 (1.516–2.131)	<0.001
**Validation cohort**				
**All-cause mortality**				
**GNRI**				
GNRI < 98	1.846 (1.304–2.614)	<0.001	1.738 (1.217–2.482)	0.002
GNRI ≥ 98	Reference		Reference	
**CONUT**				
CONUT ≤ 1	Reference		Reference	
CONUT ≥ 2	2.032 (1.406–2.939)	<0.001	1.888 (1.289–2.766)	0.001

### Subgroup analysis

**FIGURE 3:**
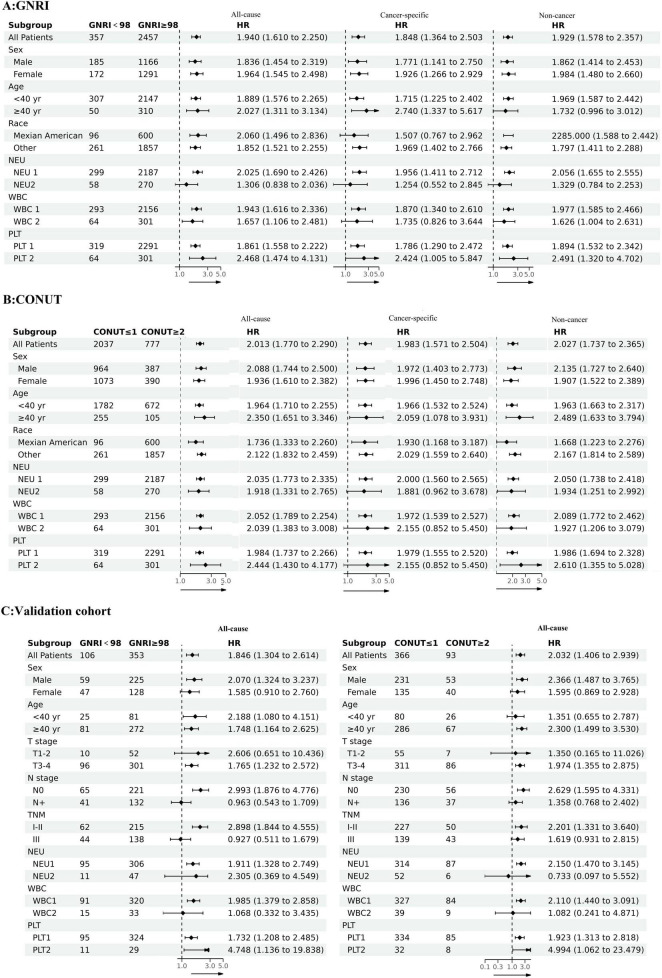
The associations between GNRI and CONUT and mortality risks in different groups **(A)** GNRI and all-cause mortality, cancer-specific mortality, non-cancer mortality; **(B)** CONUT and all-cause mortality, cancer-specific mortality, non-cancer mortality; **(C)** GNRI, CONUT and all-cause mortality in the validation cohort.

To evaluate the universality and robustness of our findings across diverse populations, we performed subgroup analyses in the development cohort using clinically relevant stratifications: age (40 as the cutoff value), gender, race, neutrophil count, platelet count, and white blood cell count. Notably, the validation cohort analysis incorporated additional clinicopathological parameters including T stage, N stage, and TNM stage system, though racial stratification was omitted due to demographic homogeneity in this group. The resulting forest plots demonstrated consistent prognostic performance of both GNRI and CONUT across all evaluated subgroups (Figure 3). This concordance between development and validation cohorts reinforces the stability of our predictive models and confirms the clinical applicability of these nutritional indices.

### Association between composite stratification groups and mortality risk and survival status

To explore the predictive ability of the composite nutritional indicators, we divided patients into three risk subtypes based on different GNRI and CONUT nutritional statuses: high-risk: GNRI < 98 and CONUT ≥ 2, with the poorest nutritional status; low-risk: GNRI ≥ 98 and CONUT ≤ 1, with the best nutritional status; and the remaining patients were classified as moderate-risk. The baseline characteristics are showed in Table 1.

Univariate analysis results showed that the three risk subtypes in the composite stratification groups were risk factors affecting long-term prognosis, and this result was consistent in both the development and validation groups, with survival differences visually represented by Kaplan-Meier curves (Figure 2).

After multivariate analysis, compared with the low-risk group, the risk of all-cause mortality increased by 212.7% (95%CI = 2.460–3.976, *P* < 0.001) in the high-risk group of the development cohort, and the risk of cancer-specific mortality increased by 282.3% (95%CI = 2.594–5.633, *P* < 0.001), while the risk of non-cancer mortality increased by 179.7% (95%CI = 2.054–3.809, *P* < 0.001). In the validation cohort, the risk of all-cause mortality increased by 105.0% (95%CI = 1.290–3.257, *P* = 0.002) in the high-risk group, and by 86.2% (95%CI = 1.241–2.792, *P* = 0.003) in the moderate-risk group (Table 3).

**TABLE 3:** COX proportional hazard regression analysis for composite stratification groups.

Development cohort				
All-cause mortality				
Variables	Univariate analysis		Multivariate analysis	
Composite stratification groups	HR (95%CI)	*P*	HR (95%CI)	*P*
High-risk	3.358 (2.689–4.193)	<0.001	3.127 (2.460–3.976)	<0.001
Moderate-risk	1.806 (1.580–2.063)	<0.001	1.700 (1.475–1.959)	<0.001
Low-risk	Reference		Reference	
**Cancer-specific mortality**				
High-risk	3.711 (2.566–5.366)	<0.001	3.823 (2.594–5.633)	<0.001
Moderate-risk	1.538 (1.198–1.974)	<0.001	1.578 (1.217–2.047)	<0.001
Low-risk	Reference		Reference	
**Non-cancer mortality**				
High-risk	3.169 (2.398–4.188)	<0.001	2.797 (2.054–3.809)	<0.001
Moderate-risk	1.930 (1.648–2.260)	<0.001	1.742 (1.469–2.066)	<0.001
Low-risk	Reference		Reference	
**Validation cohort**				
**All-cause mortality**				
High-risk	2.381 (1.522–3.724)	<0.001	2.050 (1.290–3.257)	0.002
Moderate-risk	1.771 (1.199–2.616)	0.004	1.862 (1.241–2.792)	0.003
Low-risk	Reference		Reference	

## Discussion

This study systematically explored the prognostic value of composite nutritional indicators, especially the Geriatric Nutritional Risk Index (GNRI) and Controlling Nutritional Status (CONUT) score, for mortality risk stratification in early - onset (EO) cancer survivors. Our findings build on prior evidence from other cancer cohorts, emphasizing nutritional status as a key determinant of oncological outcomes (13–15).

We align with pan - cancer evidence on the prognostic utility of GNRI and CONUT. For instance, in hepatocellular carcinoma, Kanno et al. showed GNRI < 98 linked to poor post - hepatectomy survival; (16) Wang et al. (17) confirmed its prognostic role in metastatic non-small cell lung cancer, (17) and Xiang et al. (18) extended this to EO colorectal cancer, showing a link between lower GNRI and advanced tumor stage (18). The CONUT score, with its multidimensional assessment of nutritional, immunological, and inflammatory biomarkers, offers unique prognostic insights. Soraya Kheirouri found a high preoperative CONUT an independent prognostic factor for overall and cancer - specific survival in pan - cancer patients, superior to the prognostic nutrition index (15) in gastric cancer, Kuroda et al. (19) found CONUT an independent survival predictor after curative resection; (19) Furthermore, multiple studies have demonstrated that the CONUT score is significantly associated with critical oncological outcomes, including postoperative complications, tumor stage, and progression-free survival (20–22). These studies support CONUT’s prognostic value for cancer patients.

Both GNRI and CONUT involve serum albumin concentration, which is closely related to inflammatory responses. Low albuminemia is a systemic inflammatory response, and inflammatory cytokines such as IL-6 reduce hepatic albumin synthesis and its mRNA content (23). Weight loss is also considered a systemic response for cancer patients. From this perspective, we believe that the predictive effect of GNRI, which combines weight and serum albumin concentration, is better than using serum

albumin alone. Additionally, CONUT uses serum cholesterol levels, and previous studies have discussed the correlation between serum cholesterol levels and cancer survival rates from multiple angles, such as intracellular signaling pathways, body energy reserves, and cancer-related protein concentrations, providing a more comprehensive perspective for CONUT assessment. Lymphocytes play an important role in immunity and nutrition, and lymphopenia is common in advanced cancer patients and is significantly associated with high aggressiveness, later stage, and worse survival in cancer patients (24, 25). Supported by these research findings, the combination of GNRI and CONUT has achieved better screening and predictive effects, enabling more precise patient stratification.

Critically, unlike simple malnutrition, cancer-associated malnutrition represents a complex pathophysiological process driven by multiple factors including reduced food intake and metabolic derangements (26). Within this spectrum, cancer cachexia constitutes a distinct hypermetabolic subtype induced by tumor-driven inflammation (27, 28). This is particularly relevant to our findings in early-onset cancer survivors, whose aggressive tumor biology and heightened metabolic demands may accelerate the transition from simple malnutrition to cachexia (8). The GNRI and CONUT—through incorporating albumin (a negative acute-phase reactant) and lymphocytes (markers of immune competence)—effectively capture this inflammatory-immune-nutritional derangement, thus explaining their superior prognostic value compared to isolated nutritional parameters.

Furthermore, in cancer patients, weight loss, impaired physical function, and systemic inflammation independently correlate with poor prognosis, (29) increased treatment toxicity leading to dose reduction/discontinuation, and reduced quality of life (26). Given the adverse tumor outcomes, significant economic burden, and heightened health demands of early-onset cancer populations, (30, 31) nutritional therapy constitutes a crucial clinical intervention. GNRI and CONUT serve as simple, accessible screening tools for early identification of malnourished patients, enabling timely clinical interventions to optimize outcomes.

The composite GNRI-CONUT stratification system (High-risk group HR = 3.36) outperforms single-index assessments, a finding with unique clinical relevance to EO populations. This contrasts with studies in elderly patients, such as Li et al.’s (29) report that CONUT alone surpassed traditional indices like PNI in predicting prostate cancer survival (9). The superiority of our composite model in EO cancers likely reflects the complex, multidimensional nature of nutritional-immune-inflammatory dysregulation in younger, metabolically active patients, underscoring the need for integrated risk assessment tools in this subgroup.

Methodologically, this study innovatively combined the large-scale, representative NHANES database with real-world hospital validation data to ensure result reliability and universality. Notably, the high-risk group exhibited a 212.7% increased all-cause mortality risk versus low-risk patients in the development cohort, with consistent validation. This heightened risk stems from EO patients’ elevated metabolic demands, which exacerbate the malnutrition-inflammation-tumor progression cycle. Clinically, this highlights the need for early nutritional interventions—such as personalized dietary plans or enteral supplements— to disrupt this vicious cycle in high-risk individuals.

However, there are still some limitations in this study. First and foremost, the absence of cancer-specific mortality data in our hospital-based validation cohort represents a significant constraint. While we validated the prognostic utility of GNRI/CONUT for all-cause mortality, we could not confirm their performance for cancer-specific survival in this cohort. This limits direct translation of our cancer-specific mortality findings from NHANES to clinical settings. Second, as a cross-sectional population survey, NHANES lacks systematic recording of cancer diagnosis dates, treatment details, pathological results, and adjuvant therapy information. This absence limits adjustment for key prognostic confounders and may introduce residual confounding in mortality risk assessment. Third, the validation cohort data came from a single center, and the cause of death data in the validation cohort was incomplete, limiting our in-depth analysis of cancer-specific death and non-cancer death, and also affecting the extrapolation of the validation results. Fourth, our operational definition of early-onset cancer—based solely on age-at-diagnosis (< 50 years) and self-reported physician diagnosis—lacks pathological confirmation and clinical staging data. It may inadvertently include misclassified cases, potentially diluting prognostic associations. To address this, future research should prioritize multicenter prospective randomized controlled trials (RCTs) to test GNRI/CONUT-guided nutritional interventions against standard care in early-onset cancer patients, tracking clinical outcomes such as chemotherapy tolerance. Complementary longitudinal studies should integrate serial biomarker assessments, including inflammatory cytokines, metabolic regulators, and body composition indices, to refine dynamic risk stratification. Finally, subtype-specific validation in rare early-onset cancers (e.g., sarcomas, gliomas) will ensure broad clinical applicability. Additionally, while GNRI/CONUT effectively screen for malnutrition risk, they cannot specifically identify the presence of cancer cachexia. Future studies integrating body composition analysis (CT/MRI) and inflammatory biomarkers could refine phenotypic characterization.

## Conclusion

In conclusion, GNRI and CONUT have good predictive value for all-cause mortality, cancer-specific mortality, and non-cancer mortality in EO cancer survivors. The composite nutritional indicators GNRI and CONUT provides a more powerful tool for nutritional assessment. Its clinical implications underscore the necessity for early implementation of malnutrition risk screening in EO cancer patients to precisely characterize their inflammation-immune-nutrition status, facilitate the formulation of individualized therapeutic strategies, and ultimately optimize clinical outcomes.

## Data Availability

Publicly available datasets were analyzed in this study. This data can be found here: https://www.cdc.gov/nchs/.
